# The *Psy*chosis MRI *Share*d *D*ata Resource (Psy‐ShareD)

**DOI:** 10.1002/hbm.70165

**Published:** 2025-02-21

**Authors:** Paul Allen, Mariana Zurita, Rubaida Easmin, Sara Bucci, Matthew J. Kempton, Jack Rogers, Urvakhsh M. Mehta, Philip K. McGuire, Stephen M. Lawrie, Heather Whalley, Ary Gadelha, Graham K. Murray, Jane R. Garrison, Sophia Frangou, Rachel Upthegrove, Simon L. Evans, Veena Kumari, the Psy‐ShareD Partnership

**Affiliations:** ^1^ Department of Neuroimaging, Institute of Psychiatry, Psychology and Neuroscience King's College London London UK; ^2^ WILL Chair PSY Team Centre LilNCog, INSERM U‐1172 Lille Haute de France France; ^3^ Department of Psychosis Studies, Institute of Psychiatry, Psychology and Neuroscience King's College London London UK; ^4^ Institute for Mental Health University of Birmingham Birmingham UK; ^5^ Department of Psychiatry, National Institute of Mental Health and Neuro‐Sciences (NIMHANS), Bangalore, India & Consciousness Studies Programme National Institute of Advanced Studies (NIAS) Bangalore India; ^6^ Department of Psychiatry University of Oxford Oxford UK; ^7^ Division of Psychiatry, Centre for Clinical Brain Sciences University of Edinburgh Edinburgh UK; ^8^ Schizophrenia Program, Department of Psychiatry, Escola Paulista de Medicina Universidade Federal de São Paulo (PROESQ‐EPM/UNIFESP) São Paulo Brazil; ^9^ Department of Psychiatry University of Cambridge Cambridge UK; ^10^ Department of Psychology University of Cambridge Cambridge UK; ^11^ Department of Psychiatry Icahn School of Medicine at Mount Sinai New York New York USA; ^12^ Djavad Mowafaghian Center for Brain Health University of British Columbia Vancouver Canada; ^13^ School of Psychology, Faculty of Health and Medical Sciences University of Surrey Guildford UK; ^14^ Department of Life Sciences, College of Health, Medicine and Life Sciences Brunel University of London London UK

## Abstract

Neuroimaging research in the field of schizophrenia and other psychotic disorders has sought to investigate neuroanatomical markers, relative to healthy control groups. In recent decades, a large number of structural magnetic resonance imaging (MRI) studies have been funded and undertaken, but their small sample sizes and heterogeneous methods have led to inconsistencies across findings. To tackle this, efforts have been made to combine datasets across studies and sites. While notable recent multicentre initiatives and the resulting meta‐ and mega‐analytical outputs have progressed the field, efforts have generally been restricted to MRI scans in one or two illness stages, often overlook patient heterogeneity, and study populations have rarely been globally representative of the diversity of patients who experience psychosis. Furthermore, access to these datasets is often restricted to consortia members who can contribute data, likely from research institutions located in high‐income countries. The Psychosis MRI Shared Data Resource (Psy‐ShareD) is a new open access structural MRI data sharing partnership that will host pre‐existing structural T1‐weighted MRI data collected across multiple sites worldwide, including the Global South. MRI T1 data included in Psy‐ShareD will be available in image and feature‐level formats, having been harmonised using state‐of‐the‐art approaches. All T1 data will be linked to demographic and illness‐related (diagnosis, symptoms, medication status) measures, and in a number of datasets, IQ and cognitive data, and medication history will also be available, allowing subgroup and dimensional analyses. Psy‐ShareD will be free‐to‐access for all researchers. Importantly, comprehensive data catalogues, scientific support and training resources will be available to facilitate use by early career researchers and build capacity in the field. We are actively seeking new collaborators to contribute further T1 data. Collaborators will benefit in terms of authorships, as all publications arising from Psy‐ShareD will include data contributors as authors.

## Graphical abstract

The Psy‐ShareD database will host pre‐existing structural MRI data collected at different sites across Europe, North, Central and South America, Asia and Australia that will be suitable for region of interest, voxel‐based morphometry, cortical thickness and surface area analytical approaches.

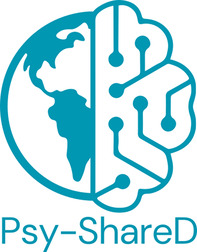

## 1 Introduction

Despite decades of research investment, schizophrenia (SCZ) spectrum disorders are still a leading cause of disability (Lopez et al. 2006; Chong et al. 2016), representing a huge economic health burden and causing untold suffering for patients and families. Given the societal and economic costs of psychotic illness and the limited efficacy of current treatment options, it is of high strategic importance that we improve our understanding of the neurobiological mechanisms that underlie SCZ spectrum disorders, to inform better detection and stratification and treatment development. Over recent decades neuroimaging research in SCZ and psychosis populations has sought to investigate neuroanatomical markers relative to healthy control (HC) groups. Numerous magnetic resonance imaging (MRI) studies investigating brain structure in SCZ and psychosis risk populations (i.e., clinical risk, familial risk and schizotypy (SZT)) have been funded in the United Kingdom and internationally, progressing our understanding of the neuroanatomical basis of SCZ and of these psychosis risk populations. The first neuroimaging anatomical investigation of patients with SCZ, using CT scans, was published in 1976 (Johnstone et al. 1976), and at the time of writing the current paper, a PubMed search using the terms ‘schizophrenia MRI (volume or thickness)’ shows that 3241 publications reporting the neuroanatomical basis of SCZ using MRI were published between 1986 and December 2024. Many structural MRI studies have also been published in first episode and psychosis risk populations. However, some single‐site MRI studies have small sample sizes (Button et al. 2013) due to the high costs of MRI, alongside difficulties recruiting patients with SCZ and other psychotic illnesses. In particular, studies in psychosis risk groups often have sample sizes under *N* = 50 (Luna et al. 2022) due to difficulties in recruiting participants meeting risk criteria. While the field has advanced significantly in recent decades and now recognises the need for enhanced sample sizes, reproducibility issues persist in the literature (Marek et al. 2022) hampering our ability to develop a definitive model of the neuroanatomical basis of SCZ, and the illness's developmental trajectory. Larger samples not only increase power to detect smaller effect sizes, but also allow studies to address the issue of clinical heterogeneity within SCZ (e.g., Chand et al. 2020), which has seriously impeded efforts to uncover a ‘common’ underlying pathophysiology (Orsolini et al. 2022). Although recent neuroimaging work has begun to address this, through clustering and subgroup analyses (e.g., Jiang et al. 2023; Alkan and Evans 2022; Chand et al. 2020), single‐site studies often lack sufficient subgroup numbers to draw reliable inferences, and the larger‐scale, multi‐site analyses have tended to just appraise between‐group differences, rather than attempting to identify neuroanatomical signatures associated with the distinct symptom profiles that exist within patient populations. Increased sample sizes allows profile‐specific inferences to be generated and confirmed through cross‐validation, allowing better translation into potential clinical benefit.

In an attempt to address this problem, there have recently been a number of notable large, international multi‐site MRI initiatives and consortia focusing on individuals at high clinical risk for psychosis or those with a diagnosis of SCZ, such as EU‐GEI (Modinos et al. 2020), PRONIA (Rosen et al. 2021), PSYSCAN (Tognin et al. 2020) and PHENOM (Chand et al. 2020). Additionally, there have been meta and mega analyses of existing data by the ENIGMA consortium (ENIGMA Clinical High Risk for Psychosis Working Group 2021; Lamsma et al. 2024; van Erp et al. 2018). While these have significantly advanced the field by improving our understanding of the anatomical changes seen in SCZ and psychosis risk populations, as well as addressing power issues, these consortia studies mostly have an over‐representation of samples from sites based in European, Australian and North American regions. While some initiatives (notably ENGIMA) have sought to improve their global reach, there is (particularly in recent years) an emergence of relevant research from sites based outside of these regions. It would be highly beneficial to incorporate such data (even if they are currently limited in number), as the paucity of samples from diverse ethnoracial groups (Fonseca et al. 2021) prevents conclusions as to whether patients from different backgrounds may show distinct patterns of neural markers linked to risk and aetiology (Fearon et al. 2006; Hutchinson et al. 1999; Kalra et al. 2012; Lim et al. 2011; Suhail and Cochrane 2002). Importantly, there is also a lack of open access neuroimaging data in psychosis: those that exist (e.g., http://schizconnect.org/) are severely limited in terms of size, global reach and what data and support are available to researchers. Although there are exceptions, such as ENIGMA, most consortia initiatives restrict data access to contributing sites and members (or do not publicise procedures for non‐contributors to access data), making it difficult for aspiring, but poorly resourced researchers outside these consortia to conduct well‐powered analyses. Thus, despite the considerable cost and effort associated with the acquisition and analyses of MRI datasets in SCZ spectrum disorder, first episode psychosis (FEP), and psychosis risk populations, clarity around the role of key brain regions and how these evolve across the disease trajectory and their link to symptoms, is still lacking (Alnæs et al. 2019; Honea et al. 2005). To address this, the ENIGMA consortium includes working groups focusing on clinical high risk (CHR) and SZT populations, and these have provided useful insights from their respective populations (e.g., ENIGMA Clinical High Risk Working Group et al. 2021; Kirschner et al. 2022). However, the field would benefit from a neuroimaging resource within which all disease phases along with corresponding clinical and cognitive profiles are adequately represented, to allow analyses across multiple disease phases. Additionally, as mentioned above, large‐scale analyses have to date often ignored the diverse clinical syndromes within SCZ; it is important to consider distinct symptom profiles. Together, these issues have limited our ability to draw useful inferences regarding underlying stage‐specific neuroanatomical changes and mechanisms and understand how these contribute to disease progression (Keshavan et al. 2020). Finally, many consortia outputs have relied on meta‐analyses of summary data from contributing studies/centres (although some of the recent ENIGMA outputs have been based on mega analyses of pooled subject data (Lamsma et al. 2024)). While there have been some efforts to effectively harmonise feature‐level data (e.g., cortical thickness and surface area measures) across participating centres, and most consortia do implement at least some harmonisation procedures, voxel level analyses have been more difficult to achieve until more recently (Si et al. 2024).

Another significant obstacle to progress in the field is the limited access to MRI datasets within SCZ and psychosis populations, which remains largely confined to a select group of researchers. In the United Kingdom, for instance, only a few centres have collected and published MRI data on SCZ populations. The study of psychosis risk cohorts is even more concentrated, with research primarily conducted at centres in London, Cambridge, Birmingham (for CHR) and Edinburgh (for familial high risk). This situation underscores the need to promote greater equity in scientific research, aligning with the UK government's ‘levelling up’ agenda. The current inequity has hindered the field's ability to pursue essential new research, address existing knowledge gaps and resolve inconsistencies. Additionally, early career researchers often face challenges in accessing these MRI datasets, limiting their opportunities for development and research, which in turn weakens overall research capacity in this critical area.

Therefore, the Psychosis Shared MRI Data Resource (Psy‐ShareD) is important for the following key reasons: First, although the prevalence of SCZ is uniform globally (Jongsma et al. 2018), there are subtle variations in the illness characteristics, including long‐term outcomes, across geographies and ethnocultural spaces (see Kalra et al. 2012 for review). Second, many brain‐based investigations into the pathogenesis of SCZ have been conducted in the Global North and other developed countries, while a majority of the SCZ populace resides in the Global South (Kalra et al. 2012). Lastly, there is a growing literature on how ethnicity (encompassing genetic, linguistic, cultural and environmental factors) can impact brain structure and function (Gong et al. 2015; Strawbridge et al. 2018).

## 2 Psychosis Shared MRI Data Resource (Psy‐ShareD) and Methods

### 2.1 Agents

For clarity going forward, here we define terms for the following agents and groups that are used in the sections below.

- *The Psy‐ShareD Partnership* comprises *Data Contributors* and *Team Members*.
- *Data Contributors* are those agents that have contributed MRI and linked clinical, demographic and cognitive data to Psy‐ShareD.
- *Team Members* are those agents who are co‐investigators and project staff. *Team Members* can also be *Data Contributors*.
- *Data Users* are those agents that access Psy‐ShareD data for analyses and publication. *Data Users* can also be *Data Contributors* and *Team Members*.

### 2.2 Aims and Objectives

The *Psy‐ShareD Partnership* is funded by the UK Medical Research Council (MR/X010651/1) with the remit to combine high‐quality, pre‐existing ‘legacy’ structural MRI datasets with linked clinical and cognitive data into one free‐to‐access resource. Psy‐ShareD is suitable for a priori hypothesis testing, exploration for novel hypothesis generation and methodological training. Specifically, the *Psy‐ShareD Partnership* brings together pre‐existing structural MRI scans in people with SCZ, FEP, CHR for psychosis, SZT and HC populations. The resource also includes MRI data from people diagnosed with mood disorders, notably major depressive disorder (MDD) and bipolar disorder (BPD). The latter populations, although not belonging to the psychosis spectrum, are diagnostic groups we plan to expand in the future (see Section 4). The resource will also contain measures of childhood trauma (CT) in both clinical and HC populations, potentially allowing categorical and correlational type analyses examining the effects of early experiences on neuroanatomy. Overall, Psy‐ShareD will allow comparisons among various illness phenotypes and across illness stages, enhancing our capacity to understand both the neuroanatomical basis *and their* trajectory of SCZ and psychosis. A full list of available datasets can be viewed on the Psy‐ShareD website (https://psyshared.com/Data.html).

To summarise, the primary objectives of the *Psy‐ShareD Partnership* are as follows:

1. Build a sustainable free‐to‐access structural MRI data repository from pre‐existing MRI datasets in SCZ, FEP, CHR, SZT, BPD, MDD and HC participants, and include within the database linked demographic, clinical and IQ data. Neuropsychological, medication, treatment responsiveness and functional outcome measures will also be included where available.
2. Seek out existing MRI datasets from across the world, particularly those acquired in non‐Western populations.
3. Organise and curate all Psy‐ShareD datasets and catalogue these clearly via Kings College London (KCL) FigShare (https://kcl.figshare.com) and the Psy‐ShareD Website (https://psyshared.com).
4. Undertake and publish a series of validation and proof‐of‐concept analyses using Psy‐ShareD datasets to demonstrate the reliability and feasibility of use.

### 2.3 Data Transfer

**FIGURE 1:**
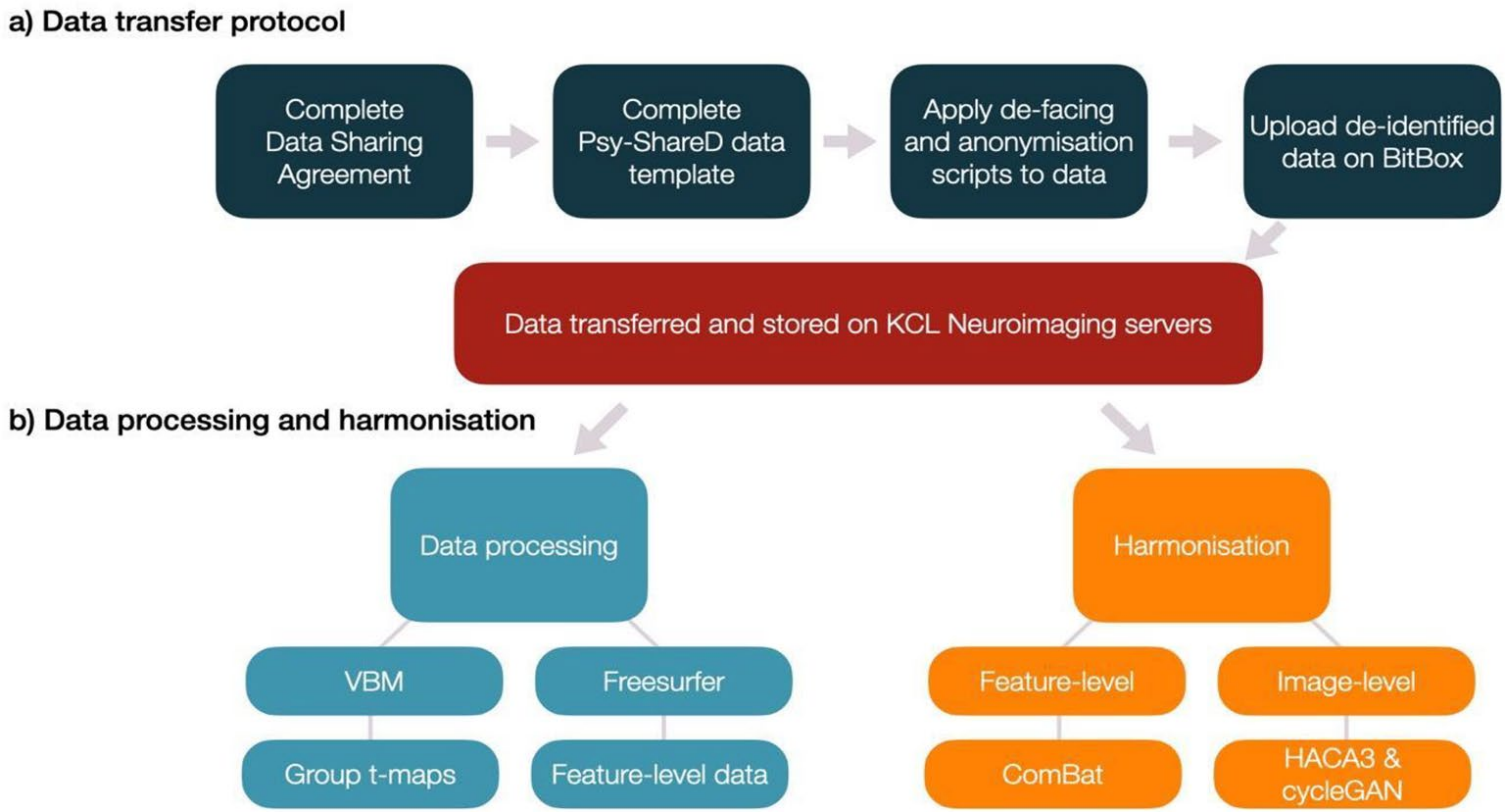
Schematic showing Psy‐ShareD procedures for (a) data transfer and preparation steps taken by the data contributors (dark blue) prior to storage (red), (b) data processing (light blue) and harmonisation (orange) steps and results carried out by the Psy‐ShareD team.

Contributing centres will share raw T1 images after defacing and removing any personally identifying information. This allows *Psy‐ShareD* to conduct rigorous harmonisation procedures based on the raw data while safeguarding participants' privacy and better accounting for variability between sites, and providing users with a higher level of reliability and power. All Psy‐ShareD partner institutions that share data complete a data sharing agreement (DSA). More information about the Psy‐ShareD DSA process are provided on our website (https://psyshared.com/Datacontribution.html). Once a DSA is established with the contributing partner/institution, and prior to T1 and linked data transfer, the *Psy‐ShareD Team* provides the transferring sites with scripts and instructions for data de‐identification (defacing) and full anonymisation, in accordance with the Psy‐ShareD Data Transfer Protocol (Figure 1). A script is also provided to produce unique Psy‐ShareD ID codes for each MRI T1 scan transferred from the contributing site. These ID codes also link MRI and corresponding clinical, demographic and cognitive data. De‐identification and anonymisation scripts are available via the Psy‐ShareD website. Once data preparation steps are completed, data are transferred via BitBox (https://www.bitbox‐imaging.com). BitBox (Easmin et al. 2022) was developed for the purpose of imaging and clinical data transfer within multi‐site studies. BitBox is used to transfer medical imaging data from external and independent sites into the KCL neuroimaging network, securely and in accordance with GDPR regulations. All datasets contained within the Psy‐ShareD resource have ethical approval for reuse and sharing. Datasets (usually older datasets) where consent for reuse cannot be traced, or that are not covered by umbrella research ethics committee approval for reuse and sharing, are not included in the resource.

### 2.4 Data Harmonisation

The T1 MRI data in Psy‐ShareD were acquired at different sites using various T1 acquisition sequences (e.g., SPGR, ADNI‐GO, MPRAGE). To account for different acquisition parameters used across sites, we harmonised the data using two types of methods: (i) feature‐level and (ii) image‐level harmonisation. To make available feature‐level data including cortical and subcortical volumes, cortical thickness and surface area data, we have used FreeSurfer version 7.3.2 (https://surfer.nmr.mgh.harvard.edu/). We then used the neuroHarmonize package (https://github.com/rpomponio/neuroHarmonize; Pomponio et al. 2020) to harmonise feature‐level MRI data by removing unwanted variation induced by scanner differences such as differences in acquisition parameters, field strength and manufacturer, while preserving biological variability between individuals using an empirical Bayes framework (Fortin et al. 2017, 2018; Johnson et al. 2007; Pomponio et al. 2020). Since this harmonisation is sample‐dependent, Psy‐ShareD tools are available to aid with feature‐level harmonisation. This allows users to harmonise feature‐level MRI data, effectively removing unwanted variation due to scanner differences while preserving the biological variability between individuals, using an empirical Bayes framework (Fortin et al. 2017, 2018; Johnson et al. 2007; Pomponio et al. 2020). Site harmonisation with the neuroHarmonize package can be applied to all or a sub‐set of the datasets in Psy‐ShareD as per users' requirements. Two advantages of neuroHarmonize over other methods (e.g., using site as a covariate in statistical models) are that it improves the removal of scanner effects in datasets with small sample sizes and does not make assumptions about the neuroimaging technique being used (Radua et al. 2020).

**FIGURE 2:**
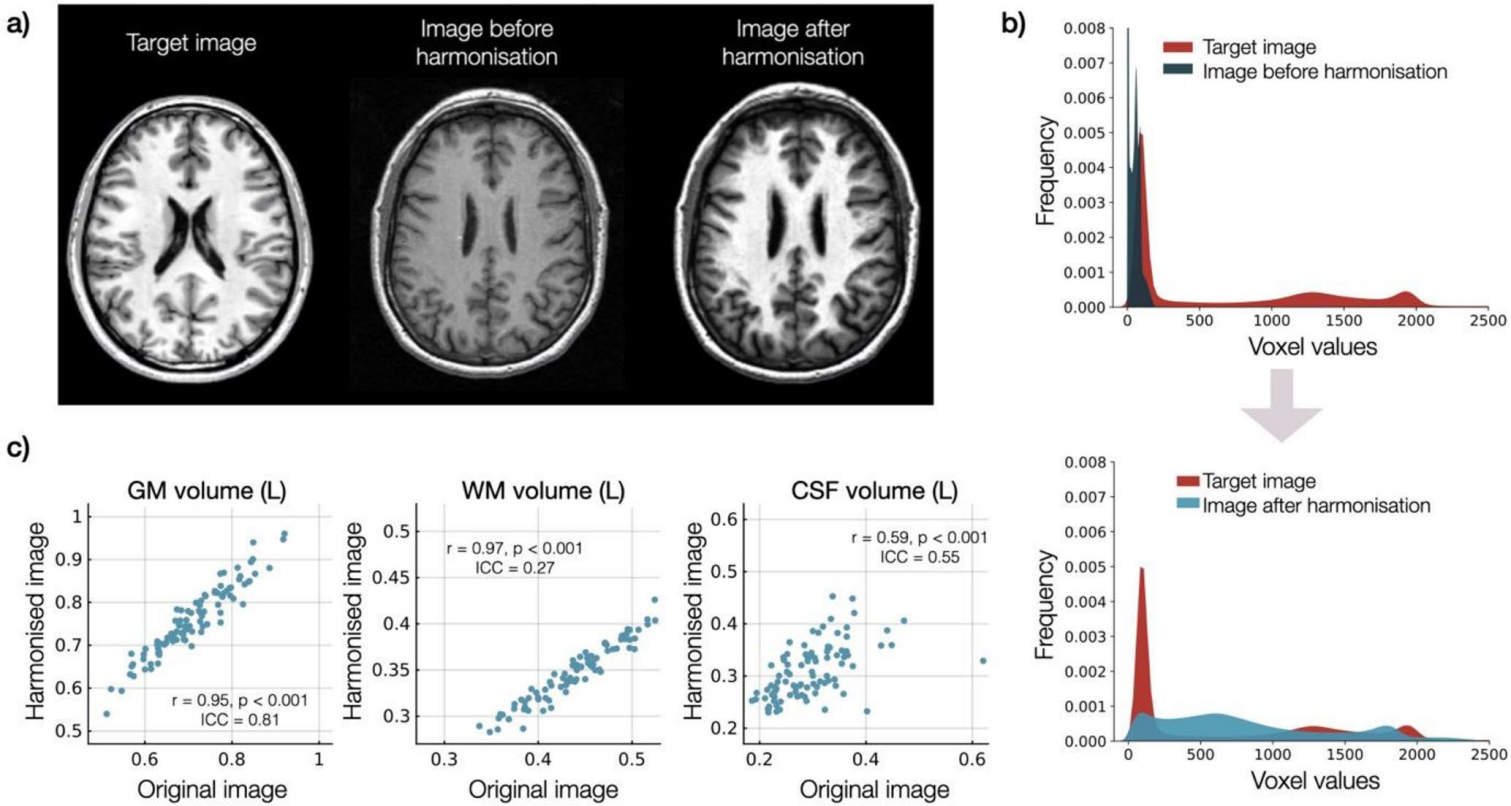
Example of harmonisation. (a) Single subject image that was harmonised towards a target image. (b) Voxel value frequency plot showing the values of voxels of the example image shown on (a) before and after it was harmonised towards a target image, (c) correlation plots showing the volumes of white matter (left), grey matter (centre) and cerebrospinal fluid (right) after segmenting several original images and their harmonised version (HACA3 image). Each plot also depicts the correlation coefficient and corresponding *p* value, as well as the intra‐class correlation coefficient (ICC(A,1) or two‐way random absolute agreement measure; McGraw and Wong 1996) between the values obtained with both images.

A unique feature of Psy‐ShareD is the use of image‐level harmonisation to T1 data, which produces whole brain images where site‐specific information is removed, therefore allowing analyses such as voxel‐based morphometry (VBM; Figure 2). We are employing two distinct methods to achieve this: HACA3 (Zuo et al. 2023) and IGUANe (Roca et al. 2025). HACA3 is a deep‐learning method with an ‘encoder‐attention‐decoder’ architecture (Zuo et al. 2023). In its simpler form, it takes two types of input images: a target image and an original input image to be harmonised towards the target image. It then encodes the anatomy, contrast and image artifact from the inputs. The method creates a synthetic image with the anatomical information from the original input image, but with the target image's contrast, while removing artifacts (Zuo et al. 2023). By harmonising an image to a target (Figure 2A), original contrast values from the original images are moved to values in the range of a target image (Figure 2B), while maintaining its anatomical features (Figure 2C). Full data validation work using these harmonisation processes is underway and will be reported separately.

IGUANe is based on the CycleGAN (Cycle Generative Adversarial Network) architecture (Zhu et al. 2017), which features generator‐discriminator pairs. The generator produces synthetic images that closely resemble the real dataset distribution, while the discriminator distinguishes between synthetic and real images. CycleGAN incorporates a cycle‐consistency constraint in its loss function to ensure that unpaired image translation and content (anatomical structure) preservation are achieved. This model is particularly beneficial for image‐level harmonisation as it requires no supervision during the training process. Since no ground truth is required for training, the output of the second GAN (generative adversarial network) must correspond only with the input of the first GAN. This means that there is no need for acquiring scans from the same participant at each site (Cackowski et al. 2021). Given that the original study focused on a 2D deep‐learning framework, we used the 3D framework of CycleGAN proposed by Roca et al. (2025), which extends the model allowing it to harmonise unseen sites. This adaptation uses PatchGAN discriminators with 3D convolutions to process entire 3D MRI scans. Scans are visually inspected, and we conduct rigorous quality checks throughout our processing pipeline to identify and address any errors. All datasets contain linked clinical, demographic and cognitive/IQ data. Where needed, harmonisation and standardisation procedures are available for these data types. The Psy‐ShareD team is currently developing tools that will allow the standardisation of clinical and cognitive variables contained within the resource using ComBat‐GAM algorithms similar to those used by the ENIGMA Clinical Endpoints Work Group (Kennedy et al. 2024) and conversion equations for clinical assessment tools (Grot et al. 2021). These tools can be adapted by data users as needed, for example, for standardising predictive variables, and so forth.

### 2.5 Data Description

All MRI T1 data are linked to anonymised demographic and clinical data as detailed in our data catalogues available via KCL FigShare (https://kcl.figshare.com). All datasets include information about participants' age and sex. Ethnoracial data, educational level and handedness are also available in many datasets. All datasets include caseness, and several datasets also include data for illness duration/onset and medication status. Symptom severity in SCZ and FEP populations was derived from the Positive and Negative Symptom Scale (PANSS; Kay et al. 1987) and the Scale for the Assessments of Positive and Negative Symptoms (SAPS/SANS; Andreasen 1984). Cohorts of individuals at high risk for psychosis were assessed for caseness and severity using the Comprehensive Assessment for an At‐Risk Mental States (CAARMS; Yung et al. 2005) and with SIPS/SOP (McGlashan et al. 2001) for some datasets. Global functioning (General Assessment of Functioning, GAF; Hall 1995) and measures for depression and anxiety symptoms are also available in several datasets. In non‐clinical risk groups (SZT and CT), a range of sub‐clinical measures are provided where available, such as the Oxford‐Liverpool Inventory for Feelings and Experiences (Mason and Claridge 2006), Schizotypal Personality Questionnaire (Raine 1991) and Childhood Trauma Questionnaire (Bernstein et al. 2003). Cognitive and IQ data are also available in several datasets assessing intellectual function, working memory and executive function and verbal learning (see data catalogues).

## 3 Psy‐ShareD Data Management: Access and Storage

### 3.1 Data Access and Storage

Data catalogues are available through KCL FigShare (https://kcl.figshare.com) and the Psy‐ShareD website (https://psyshared.com/Data.html). Data catalogues contain information about each dataset, including caseness, sample size, data formats, demographics, clinical, medication and cognitive variables.

**FIGURE 3:**
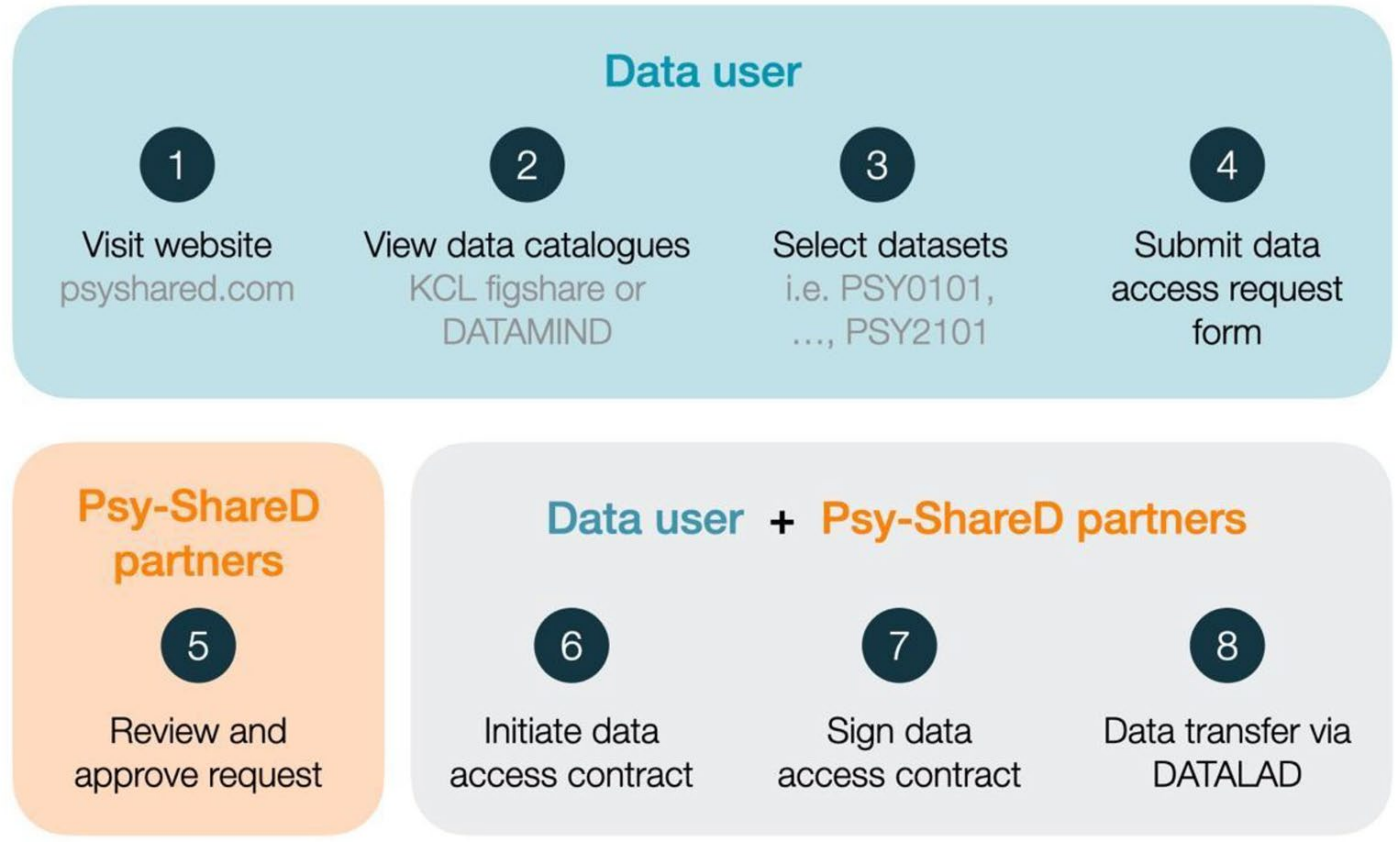
Psy‐ShareD data access procedure.

MRI T1 and linked clinical/demographic/cognitive data will be stored separately on the KCL Neuroimaging Network and will not be directly accessible to users. Members of the Psy‐ShareD Partnership act as custodians of the data contained within the resource. Potential data users can access data contained in the Psy‐ShareD database by submitting a data access request using a short data access form available on the Psy‐ShareD website. The data access procedure is shown in Figure 3. The data access procedure is required to track and monitor access and to allow data contributors whose datasets are requested to provide approval.

Once a project is approved, after the data access request, data access will be managed via DataLad and GIN (G‐Node Infrastructure). DataLad (https://www.datalad.org/) is an open‐source data management tool that tracks data and ensures reproducibility. On the other hand, GIN (https://gin.g‐node.org/) is a free and open platform for data storage and distribution, allowing data to be accessed and managed from various locations while keeping it synchronised and backed up. After the data are prepared with DataLad, it will be shared through the GIN application. This combination supports dataset versioning and provides controlled access for collaborators.

All Psy‐ShareD data users will be required to adhere to the memorandum of understanding (MoU) which can be accessed via the Psy‐ShareD website. The Psy‐ShareD MoU provides details and instructions for Psy‐ShareD *Data Users* about publication, authorship and open access data arrangements.

### 3.2 Data Use and Publication Policy

Full details are available in the Psy‐ShareD MoU document (see website). Briefly, we stipulate that all outputs that use Psy‐ShareD datasets include data contributors as co‐authors. In addition, the *Psy‐ShareD Partnership* will be listed on the author line of all publications using data from the Psy‐ShareD resource. As such, all publications and citations resulting from Psy‐ShareD will be linked to the respective data contributors. Specific publication policies (i.e., conference abstracts, posters, symposia) are listed in the Psy‐ShareD MoU document.

### 3.3 Tools, Training and Open Access

For partners that contribute data, instructions and tools for data preparation and transfer are available free upon request from the Psy‐ShareD team. Tools for T1 image‐level harmonisation using HACA3 and CycleGan and using ComBat for feature‐level harmonisation will also be available via the Psy‐ShareD website; all our processing protocols are fully documented and transparent. To facilitate the usage of Psy‐ShareD data, the Psy‐ShareD Partnership will develop and provide supporting material and workshops for potential users. These will include open access training resources for data organisation and analysis. Data sharing plans are fully in line with the UK Medical Research Council's data sharing policy and Open Science Framework principles (https://osf.io) that promote and support networking and partnership activities, enabling knowledge sharing and open access to data across institutions.

## 4 Future Directions and Psy‐ShareD Phase II

Psy‐ShareD is a growing resource with new datasets from around the world continually sought and being added on an ongoing basis. Currently, MRI datasets are available from sites in the United Kingdom, Europe, South and Central America, India and Japan. Further, DSAs are currently in progress with sites in North America and Australia as the resource continues to expand. We also plan to expand Psy‐ShareD transdiagnostically. The resource already includes MRI scans from patients diagnosed with BPD and MDD, and it is planned that MRI data from cohorts with other DSM‐5 disorders will be added to the resource in the future. It is also anticipated that, going forward, data from other MRI modalities can be added and linked to Psy‐ShareD T1 data, that is, resting state functional MRI and 1H‐magnetic resonance spectroscopy. In summary, future directions and objectives are as follows:

- General expansion to new sites, especially focusing on enhancing geographical and ethnoracial diversity, and continued refinement of MRI data harmonisation protocol (e.g., by computing a generalised reliability map by scanning a few identical participants on some of the most representative MRI scanners).
- Expanding beyond T1 datasets to multimodal MRI (i.e., resting state fMRI, MRS).
- Increasing the range of diagnoses for future clinical samples.
- Inclusion of genetic and omics data (inflammatory markers etc.) where possible.
- Development of clinical tools, using Psy‐ShareD data.

We are exploring methodological approaches that will allow Psy‐ShareD data to be used for clinical applications, for example, clustering and neurobiological stratification (Lalousis et al. 2022) and normative modelling of brain morphometry (Haas et al. 2024) approaches. Finally, we will link our data catalogues to DATAMIND (https://datamind.org.uk) and we are exploring the possibility of also linking to HDRUK (https://www.hdruk.ac.uk).

## Ethics Statement

The authors have nothing to report.

## Conflicts of Interest

P.A. has been funded by FrieslandCampina, G.K.M. consults for Leso Digital Health. Sophia Frangou (S.F.) is a deputy editor of Human Brain Mapping and a co‐author of this article. To minimise bias, S.F. was excluded from all editorial decision‐making related to the acceptance of this article for publication.

## Data Availability

The data that support the findings of this study are openly available in Psy_ShareD at https://psyshared.com/Home.html.
